# E2F3 renders an immunosuppressive tumor microenvironment in nasopharyngeal carcinoma: Involvements of the transcription activation of PRC1 and BIRC5

**DOI:** 10.1002/iid3.987

**Published:** 2023-08-28

**Authors:** Qiang Wang, Qi Yu, Yueyang Liu

**Affiliations:** ^1^ Otolaryngology & Head and Neck Center, Cancer Center, Department of Otolaryngology, Zhejiang Provincial People's Hospital, Affiliated People's Hospital Hangzhou Medical College Hangzhou Zhejiang China

**Keywords:** baculoviral IAP repeat containing 5, E2F transcription factor 3, macrophages, nasopharyngeal carcinoma, protein regulator of cytokinesis 1, tumor microenvironment

## Abstract

**Background:**

E2F transcription factors are well‐recognized oncogenic molecules, and their correlation with immune cell infiltration has recently been reported. This work studies the impacts and mechanism of E2F transcription factor 3 (E2F3) in the growth and tumor microenvironment (TME) of nasopharyngeal carcinoma (NPC).

**Methods:**

Aberrantly expressed transcription factors in NPC were screened by abundant bioinformatics analyses. Gene expression in NPC cells was analyzed by reverse transcription‐quantitative polymerase chain reaction and Western blot analyses. Malignant behaviors of NPC cells were analyzed by cell counting kit‐8, 5‐ethynyl‐2′‐deoxyuridine labeling, Transwell assays, and xenograft tumor models. TPA‐induced THP‐1 cells (macrophages) were cultured in the conditioned medium of NPC cells to mimic tumor‐associated macrophages (TAMs) in vivo, and these TAMs were cocultured with CD8^+^ T cells. Regulation of E2F3 on protein regulator of cytokinesis 1 (PRC1) and baculoviral IAP repeat containing 5 (BIRC5) was validated by chromatin immunoprecipitation and luciferase reporter assays.

**Results:**

E2F3 was highly expressed in NPC cells, and its knockdown suppressed malignant behavior and tumorigenic ability of the cells. The E2F3 knockdown condition downregulated M2 cytokines CD163 and interleukin‐10 in TAMs, which further enhanced proliferation and activation of the cocultured CD8^+^ T cells. E2F3 promoted transcription of PRC1 and BRIC5. Furthermore, PRC1 or BRIC5 upregulation in NPC cells restored the malignant properties of NPC cells, reprogrammed the TAMs to M2 phenotype, and suppressed the CD8^+^ T cell proliferation and activation.

**Conclusion:**

This work suggests that E2F3 renders an immunosuppressive TME in NPC by activating PRC1 and BIRC5. Suppression of any member involved might favor tumor elimination.

## Graphical abstract

Highly expressed E2F3 in NPC cells activates transcription of PRC1 and BIRC5, which renders an immunosuppressive TME characterized by abundant M2 TAMs to trigger the growth and metastasis of tumor cells.

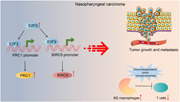

## 1 INTRODUCTION

Nasopharyngeal carcinoma (NPC) represents the most prevalent type of head and neck squamous cell carcinoma.^1^ The estimated global cases were ~133,000 and the associated deaths were ~80,000 in 2020, according to the GLOBOCAN estimates.^2^ It is a carcinoma arising from the epithelial lining of the nasopharynx, and the prevalence is particularly high in East and Southeast Asia.^3^ NPC usually presents poor prognosis attributive largely to lack of awareness of the salient symptom such as nose hemorrhage and headaches, the consequent late diagnosis, and limited therapeutic options.^4^ Radiotherapy or/and chemotherapy remain the treatment backbone for NPC, while surgery is not that applicable owing to the close proximity of nasopharynx to major blood vessels, brain stem cell area, and nerves.^4^ Increased understanding of the underpinning causative pathogenetic molecular events and identifying more therapeutic targets are crucial to enhance the patient's prognosis.

NPC tissue is heterogeneous and enriched with cancer cells, nasopharyngeal epithelium and stroma, and numerous infiltrating immune cells.^5^ Data from previous single‐cell sequencing and whole‐exome sequencing analyses indicate an immunosuppressive tumor microenvironment (TME) infiltrated by dysfunctional and exhausted CD8^+^ T cells, regulatory T cells, and M2 macrophages.^6^, ^7^, ^8^ The CD8^+^ cytotoxic T cells secret the antiproliferative, proapoptotic and antitumor interferon‐gamma (IFN‐γ) to directly destroy tumor cells.^9^, ^10^ Moreover, they can eliminate tumor cells by releasing perforin and granzymes or interacting with Fas and TRAIL receptors on tumor cells.^11^ However, in the cancer context, the M2 tumor‐associated macrophages (TAMs) in TME typically augment tumor cell proliferation, promote angiogenesis, and suppress T cell‐mediated antitumor immune response by secreting transforming growth factor‐β and interleukin‐10 (IL‐10) as well as T cell immune checkpoint ligands.^12^, ^13^, ^14^ Therefore, reprogramming the tumor‐promoting M2 type to a tumor‐inhibitory M1 type may enhance the antitumor immune response.

To identify key pathogenetic factors implicated in NPC progression, we downloaded four GSE datasets from the GEO system (https://www.ncbi.nlm.nih.gov/gds/?term) to screen aberrantly expressed genes in NPC and had them cross‐filtered with human transcription factors. E2F transcription factor 3 (E2F3) was then selected as the study subject. The E2F transcription factor family members are known to play significant roles in cell cycle progression, DNA damage repair, cell proliferation, and tumorigenesis.^15^, ^16^ Of note, these transcription factors, including E2F3, have been reportedly linked to immune cell infiltration in the TME of NPC.^17^ However, the function of E2F3 in TAM phenotype and immune response in NPC remains less known. In addition, our bioinformatics analyses identified protein regulator of cytokinesis 1 (PRC1) and baculoviral IAP repeat containing 5 (BIRC5) as two promising targets of E2F3. Intriguingly, both PRC1 and BIRC5 have been demonstrated to be linked to M2 TAM infiltration in prostate cancer.^18^, ^19^ Herein, this study aims to investigate the regulation of E2F3 on PRC1 and BRIC5 and the effects of these molecules on TAM phenotype alteration and CD8^+^ T cell activity in NPC.

## 2 MATERIALS AND METHODS

### 2.1 Cell lines and treatment

Human nasopharyngeal epithelial cells NP69 were procured from BeNa Culture Collection. NPC cell lines HNE3, C666‐1, and HK1 were procured from Chuan Qiu Biotechnology. These cells were cultured in Dulbecco's modified Eagle's medium along with 10% fetal bovine serum (FBS). Monocytic leukemia cells (THP‐1) and CD8^+^ cytotoxic T cells were procured from American Type Culture Collection. After cell recovery in Roswell Park Memorial Institute‐1640, the THP‐1 cells were induced with 12‐O‐tetradecanoylphorbol 13‐acetate (TPA) to differentiate into macrophages, and the CD8^+^ T cells were activated and expanded by CD3/CD28 magnetic beads (MBS‐C001, Acro Biosystems) stimulation. All cells were maintained in a 37°C incubator with 5% CO_2_.

### 2.2 Artificial gene alteration by lentivirus infection

Lentivirus vectors, short hairpin (sh) RNA of E2F1 (sh‐E2F3), and overexpression plasmids of PRC1 and BIRC5 (oe‐PRC1 and oe‐BIRC5) were procured from VectorBuilder Inc. In brief, the NPC cell lines were cultured in plates and treated with lentivirus solutions when a 70% confluence was reached. After 48 h, stably infected (transfected) cells were screened by the addition of the corresponding antibiotics of the lentivirus.

### 2.3 Reverse transcription‐quantitative polymerase chain reaction

Total RNA in cells was extracted by TRIzol reagent (Invitrogen, Thermo Fisher Scientific). After concentration and purity examination by microplate reader, the RNA was quantified using the SuperScript IV one‐step RT‐PCR system (Cat. no. 12594100, Thermo Fisher Scientific). Expression values of target genes relative to the internal loading gene (GAPDH) were calculated by the $2^{-{\mathrm{\Delta \Delta C}}_{t}}$ method. The primers are listed in Table 1.

**Table 1:** Primer sequences for RT‐qPCR.

	Forward primer (5ʹ‐3ʹ)	Reverse primer (5ʹ‐3ʹ)
E2F3	AGCGGTCATCAGTACCTCTCAG	TGGTGAGCAGACCAAGAGACGT
CD163	CCAGAAGGAACTTGTAGCCACAG	CAGGCACCAAGCGTTTTGAGCT
IL‐10	TCTCCGAGATGCCTTCAGCAGA	TCAGACAAGGCTTGGCAACCCA
PRC1	ATAGCCAGGAGCAGAGACAAGC	AACCGCACAATCTCAGCATCGTG
BIRC5	CCACTGAGAACGAGCCAGACTT	GTATTACAGGCGTAAGCCACCG
GAPDH	GTCTCCTCTGACTTCAACAGCG	ACCACCCTGTTGCTGTAGCCAA

Abbreviations: BIRC5, baculoviral IAP repeat containing 5; E2F3, E2F transcription factor 3; GAPDH, glyceraldehyde‐3‐phosphate dehydrogenase; IL‐10, interleukin 10; PRC1, protein regulator of cytokinesis 1; RT‐qPCR, reverse transcription‐quantitative polymerase chain reaction.

### 2.4 Western blot analysis

Total protein in cells was extracted using the cell lysis buffer (R0278, Sigma‐Aldrich, Merck KGaA), and the protein concentration was examined by the bicinchoninic acid kit (BCA1, Sigma‐Aldrich). The protein was separated by SDS‐PAGE and wet‐transferred to polyvinylidene fluoride membranes, which were blocked by 3% nonfat milk for 45 min and probed by the antibodies of E2F3 (1:500, E8651, Sigma‐Aldrich), PRC1 (1:1000, 15617‐1‐AP, Proteintech Group, Inc.), BIRC5 (1:1000, #2802, Cell Signaling Technology), and GAPDH (1:1000, #97166, Cell Signaling Technology) overnight at 4°C. The next day, the membranes were incubated with goat anti‐mouse IgG (HRP; 1:2000, ab6789, Abcam Inc.) at 20–25°C for 1 h. The protein blots were developed by enhanced chemiluminescence (WBULS0100, Sigma‐Aldrich), and the level of target protein relative to the internal loading (GAPDH) was analyzed.

### 2.5 Cell proliferation detection

The proliferation of NPC cells was analyzed following the instructions of the cell counting kit‐8 (CCK‐8; ab228554, Abcam) and 5‐ethynyl‐2′‐deoxyuridine (EdU) labeling assay (C0081S, Beyotime Biotechnology Co., Ltd.) kits. For CCK‐8, approximately 5 × 10^3^ NPC cells were cultured in 96‐well plates at 37°C with 5% CO_2_ for 0, 24, 48, and 72 h, respectively, followed by the addition of CCK‐8 reagent for 1 h of incubation. The cell viability was evaluated by reading the optical density at 450 nm read using a microplate reader. For EdU assay, approximately 1 × 10^5^ NPC cells were cultured in 24‐well plates for 48 h, followed by the addition of EdU reagent for 2 h. The cells were fixed with paraformaldehyde, penetrated with Triton X‐100, incubated with Click solution in the dark for 30 min, and then stained with Hoechst 33342 for microscopy observation.

### 2.6 Transwell assays

The mobility of the NPC cells was analyzed using Transwell chambers. In short, approximately 1 × 10^5^ NPC cells were resuspended in serum‐free medium and seeded into the upper wells, and the lower wells were filled with 10% FBS‐contained medium as attractant. For cell invasion analysis, the upper wells were pre‐coated with Matrigel. Cells in the Transwell chambers were routinely cultured for 24 h, and those migratory or invasive cells were fixed, stained with crystal violet (R23273, Saint Biotechnology), and counted.

### 2.7 Coculture of NPC cells with macrophages

The THP‐1 cells were induced with TPA for 48 h to differentiate to macrophages. The conditioned medium of NPC cells was mixed with the culture medium of macrophages (1:1) to induce TAMs, in which the expression of M2 phenotype cytokines CD163 and IL‐10 was detected by reverse transcription‐quantitative polymerase chain reaction (RT‐qPCR). The induced TAMs were cocultured with CD8^+^ T cells in 0.4‐µm Transwell plates, with TAMs seeded in the upper wells while T cells in the lower wells. After 4 days of incubation, the proliferation of CD8^+^ T cells and the expression of IFN‐γ were examined by flow cytometry. For the proliferation test, the CD8^+^ T cells were pre‐labeled carboxyfluorescein diacetate succinimidyl ester (CFSE; C34554, Invitrogen).

### 2.8 Analysis of phenotype of CD8^+^ T cells and TAMs by flow cytometry

The CD8^+^ T cells were incubated with IFN‐γ‐FITC (11‐7311‐41, Invitrogen), anti‐human CD8‐APC (#344721, BioLegend), or anti‐mouse CD8a‐APC (17‐0081‐82, Invitrogen), and TAMs were incubated with F4/80‐FITC (#23107, BioLegend) and Arg‐1‐APC (IC5868A, R&D Systems) in the dark for 30 min. The phenotype of CD8^+^ T cells and TAMs was then analyzed by flow cytometry.

### 2.9 Chromatin immunoprecipitation‐qPCR

According to the protocol of the EZ‐Magna ChIP® A kit (17‐409, Sigma‐Aldrich), the HNE3 cells were treated with formaldehyde and then with glycine to terminate the protein‐DNA cross‐linking. After that, the cells were lysed and ultra‐sonicated to truncate DNA. The lysates were incubated with anti‐E2F3 (1:50, E8651, Sigma‐Aldrich) or isotype control mouse IgG at 4°C overnight for immunoprecipitation. The protein–DNA complexes were eluted and de‐crosslinked by proteinase K. The DNA was collected and purified, in which the expression of PRC1 and BIRC5 promoter fragments was analyzed by qPCR.

### 2.10 Dual‐luciferase reporter gene assay

The binding sites of E2F3 with the PRC1 and BIRC5 promoter were predicted from hTFtarget (http://bioinfo.life.hust.edu.cn/hTFtarget/#!/prediction). Sequences containing the predicted binding sites were inserted into pGL3‐Basic vectors to construct luciferase reporter vectors. The HNE3 cells were seeded in 96‐well plates. When the cell confluence reached 70%, the cells were transfected with sh‐E2F3 along with the reporter vectors using the Hieff Trans® Liposomal Transfection Reagent (40802ES02, Yeasen Biotechnology). After 48 h, the luciferase activity in cells was analyzed by the luciferase reporter kit (11402ES60, Yeasen).

### 2.11 Xenograft tumors in mice

C57BL/6 mice were procured from Zhuhai Bestest Biotechnology Co., Ltd. and used in protocol approved by the Animal Ethics Committee of Zhejiang Provincial People's Hospital. Approximately 5 × 10^6^ HNE3 cells were resuspended in 100 µL phosphate‐buffered saline and injected into the mouse subcutaneously at the abdomen site. The long and short axes of the tumors were examined once every 5 days, and then the tumor size was evaluated as follows: Volume = 1/2 major axis × minor axis.^2^ After 1 month, the mice were killed by intraperitoneal injection of excessive nembutal (150 mg/kg) to collect and weigh the tumor tissues. Moreover, the harvested tumor tissues were made into single‐cell suspension using the tumor dissociation kit (130‐096‐730, Miltenyi Biotec), in which the TAMs and CD8^+^ T cells were sorted by F4/80 (8802‐6863‐74, Invitrogen) and CD8 (8802‐6842‐74, Invitrogen) magnetic beads.

### 2.12 Statistics

Data were analyzed by Prism 8.0 (GraphPad) and presented as mean ± standard deviation. Differences were analyzed by the *t* test (for two groups) or by the one‐ or two‐way analysis of variance (ANOVA) with Tukey's post hoc test (for over two groups). *p* < .05 was indicative of a significant difference.

## 3 RESULTS

### 3.1 E2F3 is abundantly expressed in NPC cells

**Figure 1:**
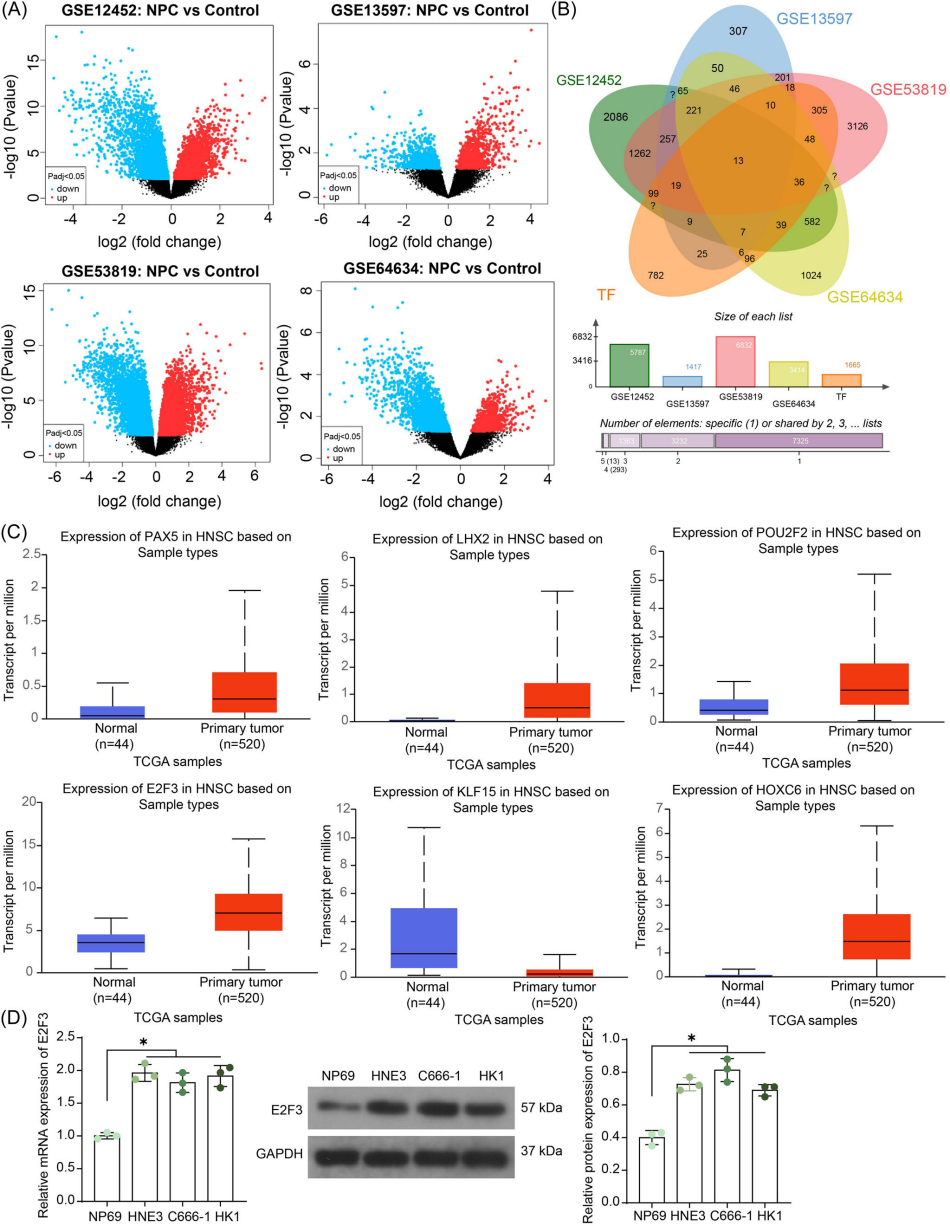
E2F3 is abundantly expressed in NPC cells. (A) Volcano plots for DE genes between NPC and control in the GSE12452, GSE13597, GSE53819, and GSE64634 datasets; (B) a Venn diagram for the intersecting factors among the DE genes and human transcription factors; (C) expression profiles of PAX5, LHX2, POU2F2, E2F3, KLF15, and HOXC6 in head and neck squamous cell carcinoma in ULACAN system; (D) E2F3 mRNA, and protein expression in NP69, HNE3, C666‐1 and HK1 cells detected by RT‐qPCR and WB analysis (*n* = 3, one‐way ANOVA). ANOVA, analysis of variance; DE, differentially expressed; NPC, nasopharyngeal carcinoma; RT‐qPCR, reverse transcription‐quantitative polymerase chain reaction; WB, Western blot. *p < .05.

Four GEO data sets (GSE12452, GSE13597, GSE53819, and GSE64634) were downloaded to analyze differentially expressed (DE) genes (*p* < .05) in NPC (Figure 1A), which were then cross‐filtered with the human transcription factors predicted from the HumanTFDB system (http://bioinfo.life.hust.edu.cn/HumanTFDB#!/download). A total of 13 candidate factors were obtained (LTF, FOXJ1, HOXC6, LHX2, NFE2L3, KLF2, HOXA10, FOXN3, E2F3, PAX5, ARNTL2, POU2F2, and KLF15; Figure 1B). Among them, six transcription factors (PAX5, LHX2, POU2F2, E2F3, KLF15, and HOXC6) have specific targets in the hTFtarget system (http://bioinfo.life.hust.edu.cn/hTFtarget/#!/). Five out of the six candidates (except LHX2) show significant differential expression in head and neck squamous cell carcinoma according to the UALCAN system (http://ualcan.path.uab.edu/index.html; Figure 1C). Among them, PAX5 has been well established to be involved in B cell development and correlated with Epstein‐Barr virus‐associated NPC,^20^, ^21^ and HOXC6 has been demonstrated to be associated with Ki‐67 expression and poor survival in NPC patients.^22^ As for POU2F2 and KLF15, there has been little evidence concerning their functions in NPC. In terms of E2Fs, they have been reportedly linked to inflammatory cell infiltration in NPC,^17^ and E2F3 in TAMs has been found to be correlated with tumor metastasis.^23^ Therefore, we conjectured that E2F3 may have specific roles in modulating TME of NPC, which is interesting and has never been investigated before. After comprehensive consideration, we selected E2F3 for subsequent research. The subsequent RT‐qPCR and Western blot (WB) analysis showed that E2F3 expression was conspicuously upregulated in NPC cell lines compared to the normal NP69 cells (Figure 1D). The HNE3 and C666‐1 cells were selected for subsequent use.

### 3.2 E2F3 knockdown blocks malignant properties of NPC cells

**Figure 2:**
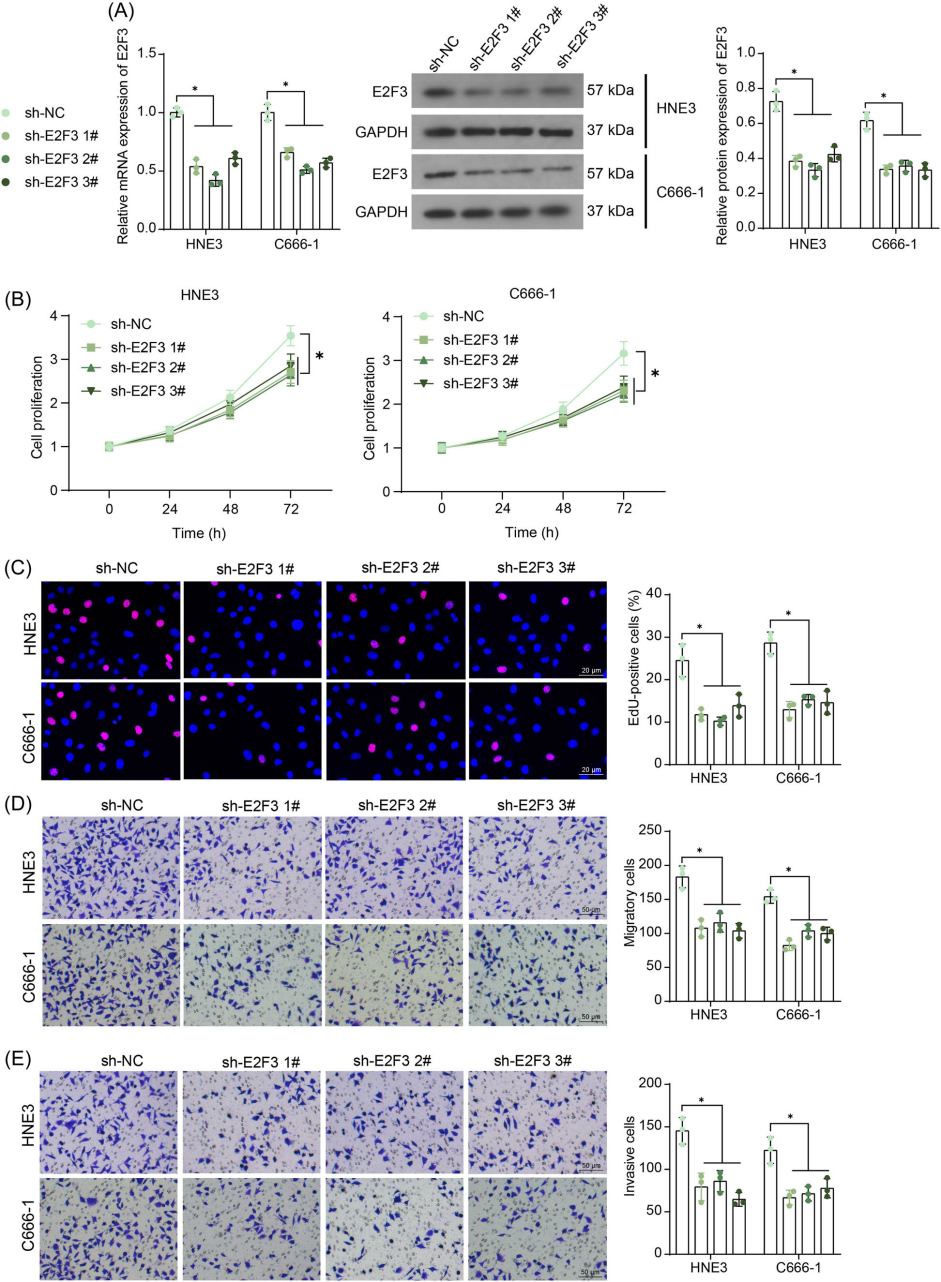
E2F3 knockdown blocks malignant properties of NPC cells. (A) E2F3 mRNA and protein expression in HNE3 and C666‐1 cells after shRNA administration determined by RT‐qPCR and WB analysis (*n* = 3, two‐way ANOVA); (B, C) viability (B) and DNA replication (C) of NPC cells determined by CCK‐8 and EdU labeling assays (*n* = 3, two‐way ANOVA); (D, E) migration (D) and invasion (E) of NPC cells tested by Transwell assays (*n* = 3, two‐way ANOVA). ANOVA, analysis of variance; NPC, nasopharyngeal carcinoma; RT‐qPCR, reverse transcription‐quantitative polymerase chain reaction; WB, Western blot. *p < .05.

Artificial downregulation of E2F3 was induced in HNE3 and C666‐1 cells using three shRNAs. Each of them suppressed the mRNA and protein levels of E2F3 in both cell lines (Figure 2A). The E2F3 silencing blocked the proliferation of the HNE3 and C666‐1 cells according to the CCK‐8 and EdU labeling assay results (Figure 2B,C). Meanwhile, it conspicuously suppressed the migratory and invasive potentials of the NPC cells (Figure 2D,E).

### 3.3 E2F3 knockdown in NPC cells reprograms phenotype of TAMs and promotes CD8^+^ T cell activation

**Figure 3:**
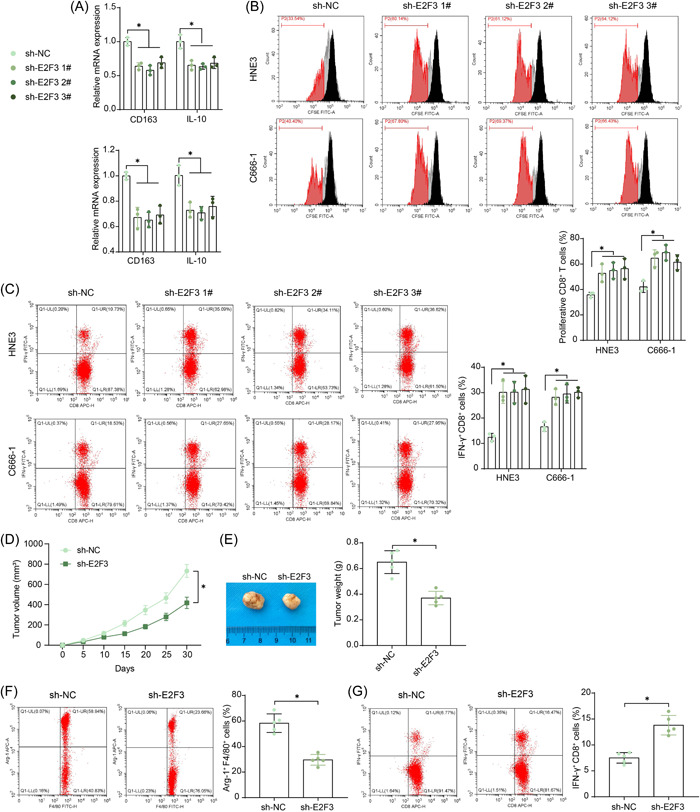
E2F3 knockdown in NPC cells reprograms the phenotype of TAMs and promotes CD8^+^ T cell activation. (A) Expression of M2 cytokines CD163 and IL‐10 in the conditioned medium (of NPCs)‐stimulated TAMs examined by RT‐qPCR (*n* = 3, two‐way ANOVA); (B, C) proliferation of (B) and the expression of IFN‐γ in (C) CD8^+^ T cells after coculture with TAMs examined by flow cytometry (*n* = 3, two‐way ANOVA); (D) growth rate of xenograft tumors formed by HNE3 cells (*n* = 5, two‐way ANOVA); (E) tumor weight on Day 30 (*n* = 5, unpaired *t* test); (F, G) infiltration of M2 TAMs (F) and CD8^+^ T cells (G) in tumor tissues analyzed by flow cytometry (*n* = 5, unpaired *t* test). ANOVA, analysis of variance; NPC, nasopharyngeal carcinoma; RT‐qPCR, reverse transcription‐quantitative polymerase chain reaction; TAM, tumor‐associated macrophage. *p < .05.

To investigate the effect of E2F3 knockdown on TAMs, we had the macrophages cultured in the conditioned medium of NPC cells to mimic the TAM condition in vivo. Of note, the E2F3 knockdown in NPC cells led to a decline in the M2 markers CD163 and IL‐10 in the TAMs (Figure 3A). These stimulated TAMs were cocultured with CD8^+^ T cells in Transwell chambers. According to the flow cytometry, the E2F3 knockdown‐stimulated TAMs increased the proliferation of CD8^+^ T cells in the coculture system (Figure 3B) and increased the proportion of activated (IFN‐γ^+^CD8^+^) T cells (Figure 3C). This evidence indicates that the E2F3 knockdown in NPC cells could suppress M2 polarization of TAMs and rescue the proliferation and activation of CD8^+^ T cells. Moreover, we injected NPC cells into mice to induce subcutaneous xenograft tumors. To reduce animal usage, only the HNE3 cell line with shRNA 2# administration (showing the best suppressive effect among three shRNAs, as shown in Figure 2A) or sh‐NC was used. The E2F3 downregulation in HNE3 cells significantly reduced the growth rate (Figure 3D) and weight (Figure 3E) of xenograft tumors in vivo. Thereafter, single‐cell suspension of xenograft tumor tissues was prepared. In the condition of E2F3 knockdown, the tissues showed reduced number of infiltrating M2 TAMs while the increased number of infiltrating CD8^+^ T cells (Figure 3F,G).

### 3.4 E2F3 downregulation suppresses transcription of PRC1 and BIRC5

**Figure 4:**
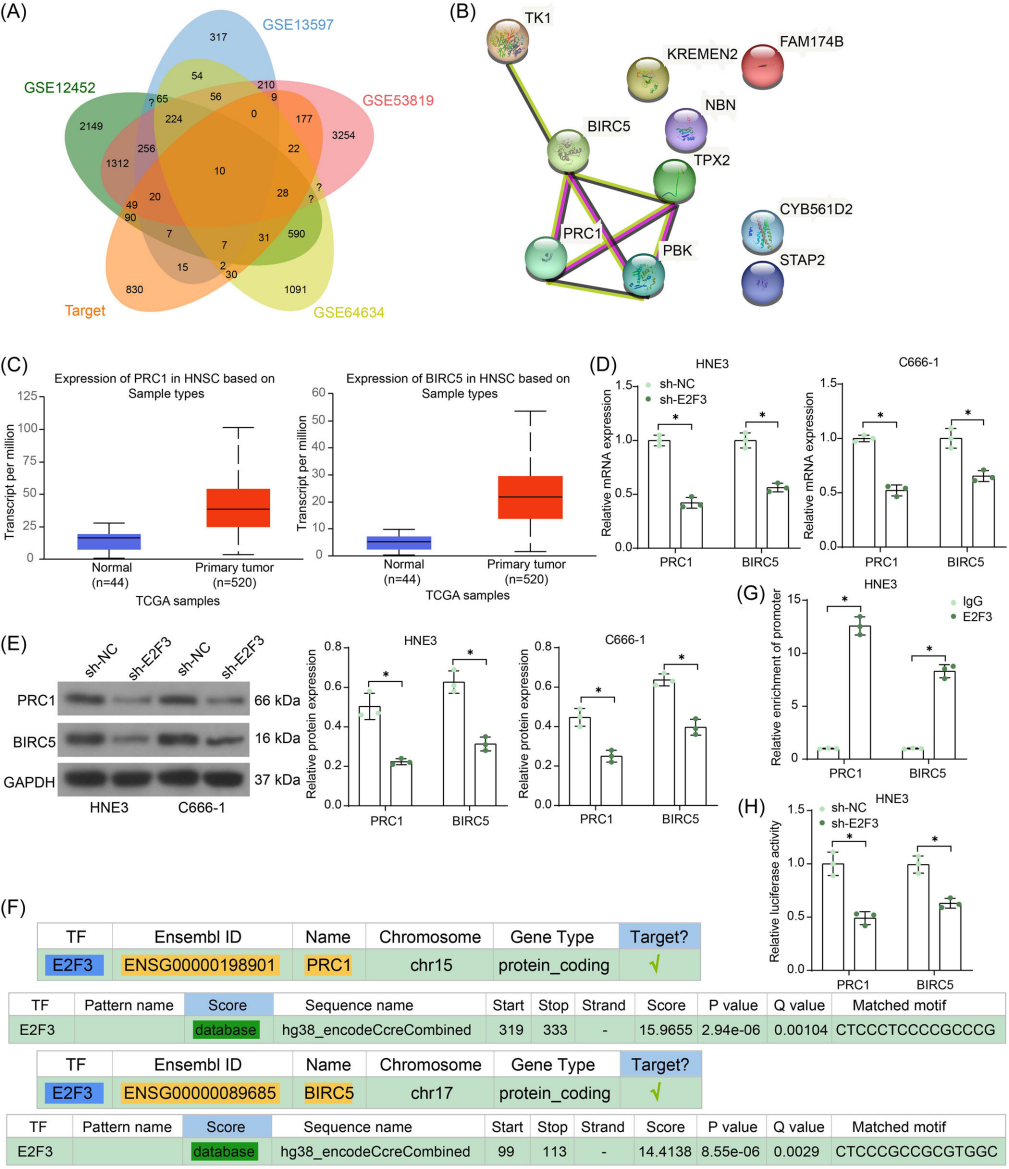
E2F3 downregulation suppresses transcription of PRC1 and BIRC5. (A) Intersections of downstream targets of E2F3 and the DE genes in the GSE12452, GSE13597, GSE53819, and GSE64634 data sets; (B) a protein–protein interaction network of the 10 intersecting factors; (C) expression profiles of PRC1 and BIRC5 in the UALCAN system; (D) mRNA expression of PRC1 and BIRC5 in sh‐E2F3‐introduced NPC cells examined by RT‐qPCR (*n* = 3, two‐way ANOVA); (E) protein levels of PRC1 and BIRC5 in sh‐E2F3‐introduced NPC cells examined by WB analysis; (F) putative binding sites of E2F3 with the promoters of PRC1 and BIRC5 predicted in the hTFtarget system; (G) PRC1 and BIRC5 promoter fragments enriched by E2F3 protein examined by the ChIP‐qPCR assay (*n* = 3, two‐way ANOVA); (H) transcriptional regulation of E2F3 on PRC1/BIRC5 examine by dual‐luciferase reporter gene assay (*n* = 3, two‐way ANOVA). ANOVA, analysis of variance; ChIP, chromatin immunoprecipitation; DE, differentially expressed; NPC, nasopharyngeal carcinoma; RT‐qPCR, reverse transcription‐quantitative polymerase chain reaction; WB, Western blot. *p < .05.

We predicted the downstream targets of E2F3 in the hTFtarget system and had them cross‐filtered with the DE genes screened from the four GEO datasets, with 10 candidates obtained (PRC1, BIRC5, TPX2, PBK, FAM174B, TK1, NBN, KREMEN2, CYB561D2, and STAP2; Figure 4A). A protein–protein interaction network of the 10 factors constructed in the STRING system (https://cn.string-db.org/cgi/input?sessionId=bEa0HT682WvW&input_page_show_search=on) shows that PRC1, BIRC5, TPX2, and PBK are the core factors (Figure 4B). For the unknown effects of PRC1 and BIRC5 in the TME of NPC, we selected these two factors for further investigation. Both PRC1 and BIRC5 were predicted to be highly expressed in NPC in the UALCAN system (Figure 4C). Of note, we observed that the mRNA and protein levels of PRC1 and BIRC5 were significantly decreased in the NPC cell lines upon E2F3 silencing (Figure 4D–E). Thereafter, we obtained the putative binding sites of E2F3 with the promoters of PRC1 and BIRC5 in the hTFtarget system (Figure 4F). Chromatin immunoprecipitation (ChIP)‐qPCR assay revealed that there were abundant PRC1 and BIRC5 promoter fragments enriched by the E2F3 protein in the HNE3 cells (Figure 4G). The dual luciferase assay also showed that the E2F3 downregulation in HNE3 cells reduced the luciferase activity of the PRC1/BIRC5 promoter‐containing reporter vectors (Figure 4H).

### 3.5 PRC1 or BIRC5 overexpression rescued malignant behavior of NPC cells

**Figure 5:**
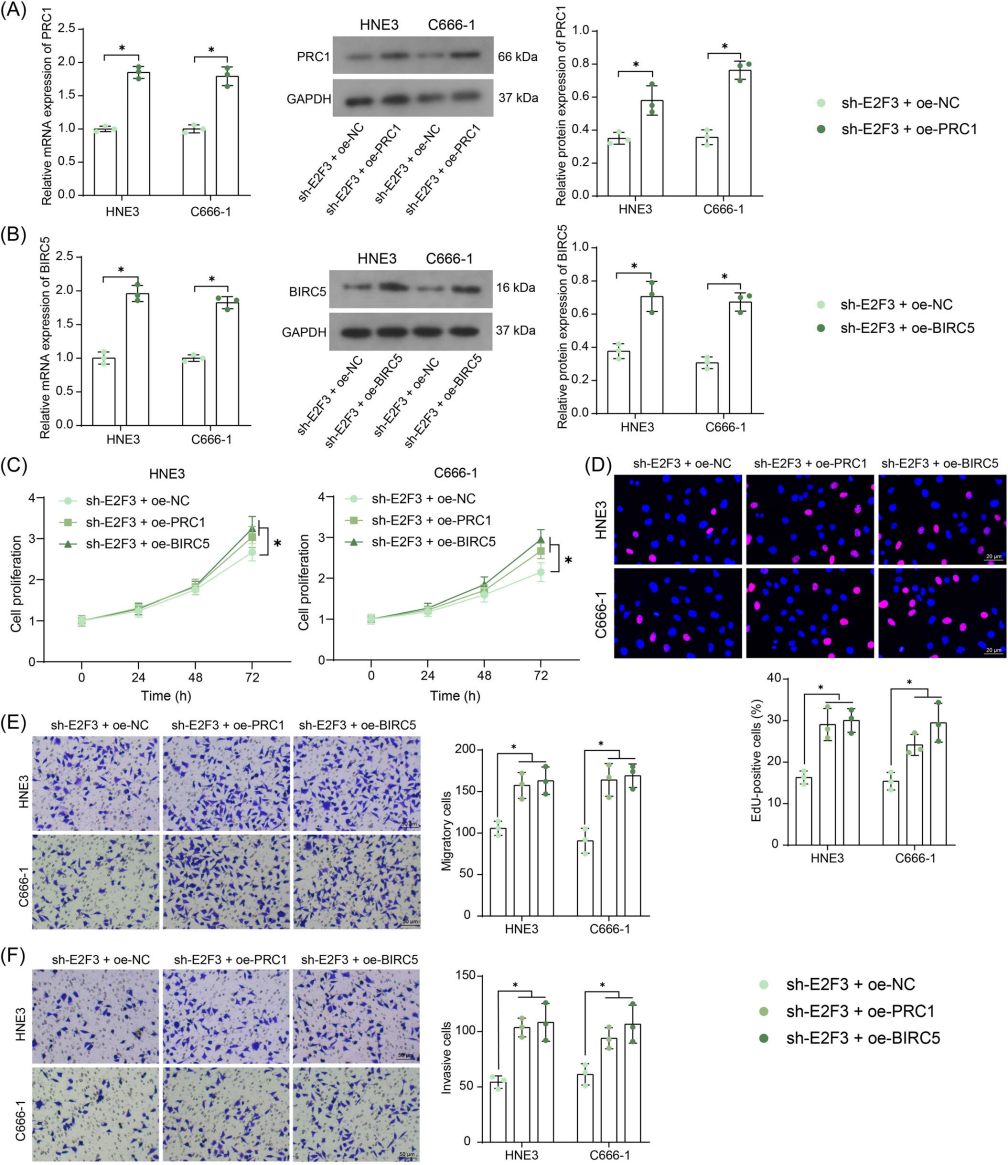
PRC1 or BIRC5 overexpression rescues the malignant behavior of NPC cells. A, mRNA and protein levels of PRC1 (A) and BIRC5 (B) in NPC cell lines examined by RT‐qPCR and WB analysis (*n* = 3, two‐way ANOVA); (C, D) viability (C) and DNA replication (D) of NPC cells determined by CCK‐8 and EdU labeling assays (*n* = 3, two‐way ANOVA); (E, F) migration (E) and invasion (F) of NPC cells tested by Transwell assays (*n* = 3, two‐way ANOVA). ANOVA, analysis of variance; CCK‐8, cell counting kit‐8; EdU, 5‐ethynyl‐2′‐deoxyuridine; NPC, nasopharyngeal carcinoma; RT‐qPCR, reverse transcription‐quantitative polymerase chain reaction; WB, Western blot. *p < .05.

The sh‐E2R3‐treated NPC cells were further administrated with oe‐PRC1 or oe‐BIRC5, and the successful PRC1 or BIRC5 upregulation in cells was detected by RT‐qPCR and WB analysis (Figure 5A,B). The PRC1 or BIRC5 overexpression promoted the viability of NPC cells (Figure 5C) and increased the number of EdU‐positive cells (Figure 5D). Meanwhile, the PRC1 or BIRC5 overexpression also promoted the migration and invasion of the HNE3 and C666‐1 cells (Figure 5E,F).

### 3.6 PRC1 or BIRC5 overexpression blocks the E2F3 silencing‐mediated immune activation

**Figure 6:**
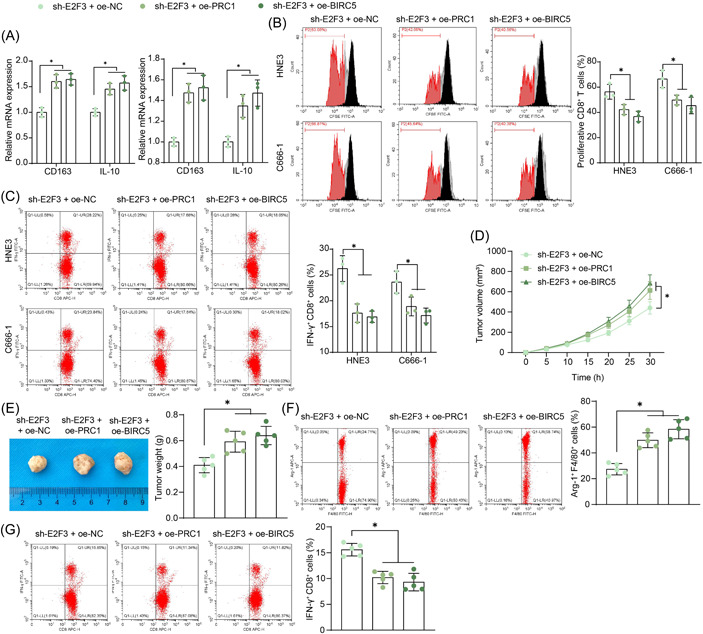
PRC1 or BIRC5 overexpression blocks the E2F3 silencing‐mediated immune activation. (A) Expression of M2 cytokines CD163 and IL‐10 in the conditioned medium (of NPCs)‐stimulated TAMs examined by RT‐qPCR (*n* = 3, two‐way ANOVA); (B, C) proliferation of (B) and the expression of IFN‐γ in (C) CD8^+^ T cells after coculture with TAMs examined by flow cytometry (*n* = 3, two‐way ANOVA); (D) growth rate of xenograft tumors formed by HNE3 cells (*n* = 5, two‐way ANOVA); (E) tumor weight on Day 30 (*n* = 5, one‐way ANOVA); (F, G) infiltration of M2 TAMs (F) and CD8^+^ T cells (G) in tumor tissues analyzed by flow cytometry (*n* = 5, one‐way ANOVA). ANOVA, analysis of variance; NPC, nasopharyngeal carcinoma; RT‐qPCR, reverse transcription‐quantitative polymerase chain reaction; TAM, tumor‐associated macrophage. *p < .05.

The conditioned medium of NPC cells with sh‐E2F3 + oe‐PRC1 or sh‐E2F3 + oe‐BIRC5 administration was collected for TAM stimulation as well. It was found that the supplementation of PRC1 or BIRC5 significantly increased the expression of CD163 and IL‐10 in the TAMs (Figure 6A). In the coculture system of TAMs and CD8^+^ T cells, the oe‐PRC1 or oe‐BIRC5‐stimulated TAMs suppressed the proliferation of T cells (Figure 6B) and reduced the proportion of activated (IFN‐γ^+^CD8^+^) T cells (Figure 6C). In vivo, the PRC1 or BIRC5 overexpression in HNE3 cells rescued the growth rate (Figure 6D) and weight (Figure 6E) of xenograft tumors. Moreover, the PRC1 or BIRC5 overexpression restored the infiltration of M2 TAMs, whereas it reduced the infiltration of CD8^+^ T cells (Figure 6F,G).

## 4 DISCUSSION

Although many TAMs existing as M1 type in the early stage of tumor are effector cells potent in disrupting tumor cells, the TME favors the transition of infiltrating TAMs to the M2 phenotype, which suppresses antitumor immune response and augments tumor progression.^24^ In the present work, we identified E2F3 as a candidate pathogenetic factor implicated in NPC development. The subsequent functional assays revealed a causative correlation of E2F3 with M2 TAM skewing, CD8^+^ T cell exhaustion, and NPC growth and development.

By cross‐analyzing the DE genes in four GEO data sets with human transcription factors and querying the UALCAN database, we obtained E2F3 as one of the five candidate transcription factors (PAX5, LHX2, POU2F2, E2F3, and HOXC6) aberrantly expressed in NPC. We focused on E2F3 as its family members have been reportedly associated with inflammatory (immune cell) infiltration and tumorigenesis in NPC.^17^ As a typical E2F transcription factor, E2F3 often recognizes a specific sequence motif in DNA and directly interacts with the retinoblastoma protein to manipulate the expression of cell cycle‐involved genes.^25^ E2F3 upregulation has been detected in several human cancer types which reportedly drives cell proliferation, differentiation, cell cycle progression, and apoptosis resistance.^25^, ^26^, ^27^ The oncogenic role of E2F3 also applies in NPC.^28^ Indeed, we validated elevated E2F3 expression in NPC cell lines and confirmed that the gene knockdown blocked proliferation, migration, and invasion, and in vivo tumorigenic ability of the cells. The specific relevance of E2F3 to immune activity in cancers has been proposed as well. For example, Liao and colleagues reported that high E2F3 expression was linked to increased infiltration of lymphocytes, macrophages, neutrophils, and dendritic cells in brain tumors.^29^ Moreover, ablation of E2F3 in TAMs has been witnessed to reduce lung metastasis of tumors.^23^ However, less has been reported concerning the role of E2F3 in TAM polarization and T cell activity. Here, we further identified that the E2F3 knockdown condition suppressed the M2 cytokines in TAMs and consequently increased the proliferation and activation of CD8^+^ T cells, suggesting an immunosuppressive role of E2F3 in NPC.

When it comes to the downstream factors involved, we predicted the downstream targets of E2F3 in the hTFtarget system and had them cross‐checked with the DE genes, with four core targets predicted (PRC1, BIRC5, PBK, and TPX2). Among them, PBK has recently been found to mediate MSL1 phosphorylation and induce the expression of CD276 (an immune checkpoint molecule) to induce immune evasion in NPC.^30^ TPX2 has also been screened as a DE gene in NPC in the study by Zou et al., but its relevance to immune cell infiltration in NPC remains untouched yet.^31^ Interestingly, PRC1 has been reported to reverse the recruitment of M2 TAMs and regulatory T cells in the TME of prostate cancer to overcome immune evasion.^19^ In hepatocellular carcinoma, likewise, high PRC1 expression has been linked to increased TAM infiltration and poor prognosis of patients.^32^ The PRC1 has also been identified as one of the hub macrophage‐related genes in colorectal cancer patients.^33^ As for BIRC5, similarly, it is reportedly expressed at particularly high levels in prostate tumors characterized by infiltration of TAMs, suggesting its potential correlation with TAM infiltration.^18^ We therefore wondered whether PRC1 and BIRC5 participate in the maintenance of M2 TAMs in the TME of NPC as well. After confirming the transcriptional regulation of E2F3 on PRC1 and BIRC5 by ChIP and luciferase assays, we performed functional assays, with the results showing that either upregulation of PRC1 or BIRC5 restored the M2 skewing of TAMs and reduced the T cell activity, along with rescued malignant properties of NPC cells.

These results, collectively, support that the aberrantly highly expressed E2F3 in NPC cells renders an immunosuppressive TME in NPC by transcriptionally activating PRC1 and BIRC5. The findings may offer novel insights into the management of NPC that blockage of E2F3, PRC1, or BIRC5 may enhance the antitumor immune response in NPC.

### 4.1 Study limitation

Due to the ethics limits, we could not obtain adequate NPC or normal tissue samples to verify the findings in clinics, which may weaken the translation value of the study. In addition, due to the fund and time limits, we only focused on the E2F3‐PRC1/BIRC5 axis in the present work. According to our bioinformatics results, there might be more molecules (such as POU2F2 and KLF15) that participate in the progression of NPC. In the same vein, there might be other molecules (such as TPX2 and PBX) that participate in the immunosuppressive events mediated by E2F3. We would like to focus on these issues in future research.

## AUTHOR CONTRIBUTIONS

**Qiang Wang**: Conceptualization; data curation; investigation; supervision; writing—original draft. **Qi Yu**: Data curation; formal analysis; methodology; supervision; writing—review and editing. **Yueyang Liu**: Methodology; project administration; resources; software; validation.

## CONFLICT OF INTEREST STATEMENT

The authors declare no conflict of interest.

## ETHICS STATEMENT

C57BL/6 mice were procured from Zhuhai Bestest Biotechnology Co., Ltd. (Guangdong, China) and used in protocol approved by the Animal Ethics Committee of Zhejiang Provincial People's Hospital.

## Data Availability

The datasets used or analyzed during the current study are available from the corresponding author upon reasonable request.
